# Are Children Severely Affected by Autism Spectrum Disorder Underrepresented in Treatment Studies? An Analysis of the Literature

**DOI:** 10.1007/s10803-018-3844-y

**Published:** 2018-12-10

**Authors:** Amy Stedman, Briana Taylor, Michael Erard, Christine Peura, Matthew Siegel

**Affiliations:** 1 Developmental Disorders Program, Maine Behavioral Healthcare, 123 Andover Road, Westbrook, ME 04092 USA; 2 Maine Medical Center Research Institute, Center for Outcomes Research and Evaluation, 509 Forest Avenue, Portland, ME 04074 USA; 3 Departments of Psychiatry and Pediatrics, Tufts University School of Medicine, 136 Harrison Avenue, Boston, MA 02110 USA; 4 Maine Medical Center Research Institute, 509 Forest Avenue, Portland, ME 04101 USA

**Keywords:** Autism, Severely affected, Communication, Cognitive

## Abstract

Despite significant advances in autism research, experts have noted that children severely affected by autism spectrum disorder (ASD) appear to have been understudied. Rigorous analysis of this observation has been limited, and the representation of severity has not been well-described. We assessed three domains of severity (communication ability, cognitive functioning, and adaptive functioning) in 367 treatment studies of children with ASD published 1991–2013. We found that the proportion of studies that included the severely affected population decreased significantly over time, as well as wide variability in measurement and reporting. Inadequate representation of the full autism spectrum in the literature could lead to an unbalanced picture of ASD and leave behind those with arguably the greatest need.

## Introduction

There has been an explosion of research in the field of autism, as evidenced by a 24-fold increase in the number of papers published on the topic over the past three decades (Chakrabarti 2017). Despite this proliferation, many experts have noted that those severely affected by autism spectrum disorder (ASD) appear to have been understudied. A recent query of the National Database for Autism Research (NDAR) revealed that just 11% of the research participants with ASD have an IQ less than 85 and that even fewer participants were categorized as minimally verbal (Jack and Pelphrey 2017). As a result, the picture of ASD that emerges from the research literature seems to be at risk of bias toward the higher functioning end of a spectrum of impairments.

This important observation—that certain subsets of the ASD population have potentially been over- or under-represented in research—has not been systematically evaluated across a broad historical range of peer-reviewed publications. While this issue may have implications for all areas of autism research, the most proximal effects may be seen in autism treatment studies, as these most directly inform current clinical practice. In this study, we set out to evaluate the degree to which the more severely affected population has been included in treatment studies, assess how severity is measured and represented, and determine what direction changes have taken, if any, over the past two decades. We focused specifically on children, for whom early treatment experiences are highly influential and therefore are heavily affected by treatment choices.

Before delving into the treatment literature, it is important to first address the fact that severity in ASD has been variably and inconsistently defined over the decades. As a result, evaluating the nature and extent of the inclusion of the severely affected end of the spectrum requires sensitivity to how severity has been defined and operationalized in the field.

Historically, autism studies have diagnosed individuals with ASD by relying on a set of core symptoms, which include restricted or repetitive behaviors and impaired social communication. However, a wide variety of incommensurate measures have been employed across treatment studies, and many do not provide specific severity ratings, use different criteria, or are not cross referenceable. While some of these tools, such as the Autism Diagnostic Observation Schedule (ADOS) (Lord et al. 2008), use a semi-structured, standardized means of diagnosis, they were not necessarily designed to provide a fine-grained look at the nuances of ASD severity or thoroughly capture the aspects of severity that may be most clinically relevant: communication ability, adaptive functioning, and intelligence. While core symptoms are useful criteria for categorical diagnoses of ASD, they are less informative for some clinical, therapeutic, and educational interventions, where the degree of language, functional, and cognitive impairment may be more relevant than the presence or absence of repetitive, restricted behaviors or social communication deficits.

In the absence of a widely-accepted, uniformly-applied definition and measurement approach for ASD severity, investigators have had several options for determining parameters of severity. One approach is to search for other potentially novel domains to measure to enhance or replace a core symptom-based definition of ASD severity. For instance, Zablotsky et al. (2015) showed that higher scores on a composite measure of family impact were more predictive of parents’ ratings of their child’s ASD severity, while descriptions of ASD symptoms in and of themselves were less predictive of parents’ severity ratings. One implication is that a significant component of severity is functional and contextual in nature. This finding should inform the field’s approach to ASD severity.

Another approach to rigorously delineating ASD severity is to employ a definition informed by prior work in the field. Enough treatment research has been published in the last several decades to serve as an adequate source of raw material for an inquiry into patterns. Over time, investigators’ efforts may be converging in a telling albeit unintended fashion, a convergence that ideally should inform what the field considers a standard of ASD severity to be. In this study, we took an approach that allowed us to see patterns in the literature and begin to derive an operational definition of ASD severity that is clinically useful.

Additionally (and perhaps as a result of the preceding), researchers and clinicians have determined the severity of patients by assessing phenotypic characteristics that are beyond the realm of core ASD symptoms. Because the clinical presentation of ASD is quite heterogeneous, a number of challenges arise when attempting to further characterize an individual along the autism spectrum. Previous research has touted the importance of identifying specific subtypes within the autism spectrum as a means of developing a framework to better characterize, research, and treat those with an ASD diagnosis (Grzadzinski et al. 2013), but if those more severely affected by ASD are not included in research, we risk missing entire subtypes. For the purposes of the present study, we chose to focus on three domains of functioning that manifest in unique ways in the ASD population and play a significant role in driving clinical treatment and educational intervention selection: cognitive functioning, communication ability, and adaptive functioning.

### Cognitive Functioning

The most recent surveillance data has indicated that around 31% of children with ASD in the United States have an IQ in the intellectual disability (ID) range (IQ ≤ 70) (Centers for Disease Control and Prevention 2018). Because of variability and inconsistency in the cognitive profile of this population, it can be common to either over- or underestimate the ability levels of a child with ASD. Verification of an ID diagnosis can allow for more targeted treatment and more specialized care in a clinical or community setting (Siegel and Gabriels 2014). Challenges to establishing a child’s cognitive abilities can be confounded by the presumption of interconnectedness of cognitive and communicative impairments. One study of minimally verbal children with ASD found that they actually exhibited a range of cognitive abilities, from profoundly impaired to average intelligence (Bal et al. 2016). These findings support the importance of primarily considering cognitive functioning and communication ability as distinct entities in the context of ASD severity.

### Communication Ability

It is estimated that approximately 30% of youth with ASD are minimally verbal (Tager-Flusberg and Kasari 2013). Core autism and related symptoms are typically more severe in minimally verbal individuals, and they often exhibit a range of challenging behaviors (Tager-Flusberg et al. 2017). There is a clear lack of consensus, however, as to how to best define ‘minimally verbal’ and measure communication ability for both clinical and research purposes. One significant barrier to this is a lack of valid and appropriate measures for directly assessing communication ability across the entirety of the autism spectrum (Tager-Flusberg and Kasari 2013). These challenges in assessment can ultimately lead to challenges in selecting appropriate treatments for this population. Researchers in the field maintain a long-term goal of developing a more robust understanding of communication ability across the autism spectrum in order to provide more comprehensive assessment and treatment of the heterogeneous autism phenotypes (Kasari et al. 2013). As such, for the domain of communication in particular, it is of significant value to better our understanding of not only *who* is represented in autism treatment studies, but *how* communication is being measured and reported.

### Adaptive Functioning

Within the context of ASD, measurement of adaptive functioning (encompassing communication, daily living skills, and socialization, among other attributes) indicates the degree of support an individual requires in their everyday life. Children with ASD are typically more delayed in their adaptive functioning than their IQ measurement would suggest (Klin et al. 2007), and these adaptive functioning delays can have significant effects on the individual’s life course. Adaptive functioning has also been found to be closely related to core ASD symptom severity. For instance, an assessment of minimally verbal school-aged children with ASD found that more severe core autism symptoms (as measured by the ADOS) were associated with more impaired adaptive functioning (Frost et al. 2017). Additionally, an assessment of children with ASD in an inpatient setting found that greater impairments in adaptive functioning and coping skills (as measured by the Vineland Adaptive Behavior Scales) were associated with an increased severity of behavioral problems (Williams et al. 2017). Further, possessing higher levels of adaptive skills has been shown to *decrease* the likelihood of psychiatric hospitalization for children with ASD (Righi et al. 2017). These associations between adaptive skills and other facets of the autism phenotype provide support for adaptive functioning being a meaningful domain through which to examine autistic severity.

Examining a spectrum disorder such as ASD demands consideration of dimensional characteristics that individually and in aggregate make up an individual. However, nowhere is the potential harm of over- or understating these characteristics greater than in treatment studies, where inadequate measurement and reporting of dimensions of autistic severity prevents clinicians and researchers from being able to define treatment group characteristics and search for clinically meaningful treatment moderators. For these reasons, in this study we focused exclusively on treatment studies.

### Mapping Representation

Previous research has begun to explore the complex landscape of autistic severity through participant representation in the scientific literature. Two early studies examined smaller subsets of the literature to broadly describe subject characteristics. Charman (1994) surveyed a decade of publications from the Journal of Autism and Developmental Disorders (JADD) and categorized each by study topic and reporting of IQ data. Eighty-one percent of the papers reported some indication of IQ, of which 74% focused on subjects with some degree of intellectual impairment (IQ < 70). The authors noted a trend over time toward providing more complete descriptive information on study subjects. While this analysis only explored one aspect of autistic severity (cognitive impairment), it provides some evidence of significant inclusion of severely-affected participants in earlier research in the field in one journal. A later study (Dunlap et al. 1999) focused specifically on treatment research and broadened the analysis of participant characteristics to publications from ten journals over an 18-year time period. While the authors found few distinct patterns, there was a notable upward trend in the proportion of research conducted in more “typical” contexts (i.e., schools, as opposed to clinical settings), suggesting that intervention research may have begun to encompass a more heterogeneous group of participants across the autism spectrum with a range in symptom severity.

Exploring more current research on the topic, Bebko et al. (2008) reviewed abstracts presented at the International Meeting for Autism Research (IMFAR) from 2004 to 2006 to examine trends in research topics, participants, and study design. Most pertinent to the present study were their findings on the reported functioning levels of study participants. Abstracts were categorized as including either “high-functioning,” “moderate/low-functioning,” or “mixed/unknown” participants based on criteria for cognitive and overall functioning. Results revealed a decrease over time in studies of moderate/low-functioning samples, despite an increase in the overall number of abstracts accepted. While this study provides some valuable insight into severity representation in autism research, the date range was limited. Furthermore, the focus on conference abstracts limited access to comprehensive descriptions of the study population that can be found in complete, published articles. However, the authors raised a number of notable concerns for future research, recognizing a trend toward less specificity in the characterization of research participants, and positing that our understanding of the autism spectrum as a whole has potential to become biased toward the higher functioning end.

Crosland et al. (2013) assessed autism intervention studies from three journals from 1995 to 2009 to examine descriptive features of both participants and setting characteristics reported as well as examine potential trends over time. General level of cognitive and adaptive functioning was categorized for each assessed study as either “typical range,” “mild to moderate disability,” or “severe to profound disability.” Level of communication ability was categorized as either “communicative,” “rudimentary,” or “nonverbal/non-communicative.” While the findings showed few trends overall, it was notable that the proportion of studies that included typically-functioning participants with autism increased over time, as measured in both cognitive and communicative functioning domains. Conversely, the percentage of studies containing participants with severe/profound cognitive functioning deficits and/or nonverbal status decreased over time. Though this analysis was limited to only three journals, it demonstrated that fewer people from the severely affected end of the spectrum were included in intervention literature over time.

Most recently, Jack and Pelphrey (2017) examined the severely affected phenotype within the context of neuroimaging research and found that those with autism and co-occurring intellectual disability, minimally verbal status, or developmental regression have been generally understudied. The authors cited methodological difficulties, as well as inconsistent definitions of severity, as contributing to this gap in the imaging research. While the scope of the review did not specifically include treatment studies, the proposed under-representation of the more severe phenotype highlighted how challenges in defining and assessing severity in the autism spectrum extend across a breadth of research areas.

### Objectives

To assess the representation of the severely affected ASD phenotype in the treatment literature, and the ways severity is conceptualized, defined, and reported, we examined the characteristics of all peer-reviewed, published treatment studies of children with ASD, excluding single case designs, through a systematic, multi-step literature search and subsequent analysis covering the period of 1991–2013. Our objectives were twofold:

1. To assess representation of the severely affected population over the past two decades in the autism literature. Due to the growing heterogeneity of the ASD population, we hypothesized that evidence of the inclusion of the severely affected in autism research would decrease over time. Rapid growth in the diagnosis of higher functioning individuals on the autism spectrum has caused the proportion of those more severely affected in the overall ASD population to decrease (Matson and Kozlowski 2011). This increased heterogeneity has thus led us to posit a similar trend within the treatment literature.
2. To examine how severity is represented in the literature as well as the criteria used to determine severity. As discussed in more detail in the “Methods” section, we have chosen to operationalize severity using three domains: communication ability, cognitive functioning, and adaptive functioning. Based on an awareness that even seminal treatment studies in the field have not always reported information on their samples’ communication ability, as well as a general lack of consensus in the field on the best measures of communication (Kasari et al. 2013), we hypothesized that communication ability would be the least frequently reported domain (relative to the cognitive and adaptive functioning domains) and have the greatest variability in the measures used to capture the domain.

## Methods

### Search Procedure

A comprehensive search was conducted in November 2013 using a previously-established EndNote library of 9408 research articles on autism. The EndNote library was originally created from an exhaustive literature search used in the development of the American Academy of Child and Adolescent Psychiatry practice parameter for the treatment of children with autism spectrum disorder (Volkmar et al. 2014) and encompassed all peer-reviewed autism research articles published from January 1991 to March 2013. Details of that search methodology are provided in the aforementioned practice parameter, but briefly that search utilized the keywords autism, autistic, rett*, asperger* or (pervasive and disorder* and develop*) to search the PubMed, PsychInfo, Cochrane, and CINAHL (EBSCO) databases for all articles pertaining to autism (Volkmar et al. 2014). That initial search yielded 13,808 abstracts, which was winnowed to the EndNote database of 9408 research articles on autism used for this study. For the current analysis an initial search of the keyword “treatment” yielded 854 results from the EndNote library, with 383 articles meeting inclusion criteria after examination of the articles’ titles and abstracts. Inclusion criteria were treatment studies with a sample size greater than 1 of pediatric subjects (< 18 years old) with an autism spectrum disorder. Studies with a sample of both children and adults were included if the majority of the subjects were less than 18 years old. Studies in which participants had multiple diagnoses were included if the majority of subjects had an ASD diagnosis. Exclusion criteria comprised studies with a sample size of one, studies with a single subject design, non-treatment studies, systematic reviews, meta-analyses, studies of adults with ASD, and studies that weren’t focused on the ASD population. Further keyword searches of “pharmacology” (210 results), “clinical trial” (46), “pilot” (60), “randomized controlled trial” (64), “single-blind” (13), “double-blind” (79), “behavioral intervention” (63), “behavior therapy” (351), “occupational therapy” (46), “speech therapy” (29), and “physical therapy” (10) yielded an additional 244 results from the endnote library that met inclusion criteria. After removal of duplicate articles, a total of 586 articles underwent full text examination, resulting in an additional 213 articles excluded, and six articles for which the full text was unobtainable, yielding a final sample size of 367 full text articles to be coded.

### Preliminary Coding

The full text of the 367 articles was examined, and the following descriptive information was extracted from each article as available: year of publication; sample size; mean age of subjects; subjects’ age range; sex of subjects; type of study (randomized controlled trial [RCT], controlled trial [CT], uncontrolled trial [UT], or case series [CS]); treatment target (i.e., outcomes such as core ASD symptoms, social skills, language and communication, etc.); and type of treatment (e.g., pharmacological, psychosocial, etc.). To assess representation of severity of the sample in each article, we extracted whether scores were reported, their values, the measures utilized and other associated information for the domains of communication ability, cognitive functioning (IQ or level of ID), and adaptive functioning.

### Severity Coding

To determine the presence or absence of severely affected patients in each study’s sample, we evaluated each article based on the predetermined operational definitions described below and the published cut-off scores for the measures utilized for each of the three severity domains (cognitive functioning, communication ability, and adaptive functioning). A research assistant with two years of experience in performing assessments with individuals severely affected by autism performed both the preliminary and secondary coding for all articles.

### Operational Definitions for Coding Severity

#### Cognitive Functioning (IQ/Level of ID) Domain

Studies that reported mean IQ scores at or below 70 were coded as having included severely affected participants. For studies that reported qualitative descriptions of intellectual impairment (e.g., moderate ID), they were coded as including the severely affected if the description indicated presence of ID in at least one subject. Studies were coded as not including the severely affected population if mean IQ was greater than 70 or if the qualitative description indicated that no study subjects had ID. Studies for which degree of severity was unable to be determined from the quantitative or qualitative reporting of measurement of ID reported were coded as “unable to be assessed.” Finally, studies that did not report any measure of IQ or ID were coded as “not reported.”

#### Communication Domain

Studies that reported mean scores that fell below the study’s measure-specific cut-offs for communication impairment were coded as having included severely affected participants. For studies that reported qualitative descriptions of impaired communication (e.g., minimally verbal, nonverbal, etc.), they were coded as including the severely affected if the description indicated the presence of impaired communication in at least one participant. Studies were coded as not including the severely affected population if mean scores fell above the measure-specific cut-offs for communication impairment or if qualitative description indicated that no study participants had impaired communication (e.g., “all fluent verbal”). Studies for which the degree of severity was unable to be determined from the qualitative or quantitative report of communication were coded as “unable to be assessed.” Studies that did not report any measure of communication ability were coded as “not reported.”

#### Adaptive Functioning Domain

Studies that reported mean scores that fell below measure-specific cut-offs for impaired adaptive functioning were coded as having included severely affected participants. For studies that reported qualitative descriptions of adaptive behavior, they were coded as including the severely affected if the description indicated the presence of impaired adaptive functioning in at least one participant. Studies were coded as not including the severely affected population if mean scores fell above measure-specific cut-offs for impaired adaptive functioning or if qualitative description indicated that no study participants had impaired adaptive behavior. Studies for which the degree of severity was unable to be determined from the qualitative or quantitative report of adaptive behavior were coded as “unable to be assessed.” Additionally, studies that did not report any measure of adaptive behavior were coded as “not reported.”

#### Overall Inclusion

If an article included the severely affected in at least one domain as detailed above, we chose a liberal definition and categorized that study as having evidence of inclusion of the severely affected in its sample. For articles with at least one domain coded as not including the severely affected and the remaining domains coded as either “unable to be assessed” or “not reported,” the study was categorized as not having evidence of including the severely affected. Finally, if all three domains were coded as “not reported,” the article’s inclusion of the severely affected was coded as “not reported.” If all three domains were coded as “unable to be assessed” the article’s inclusion of the severely affected was coded as “unable to be determined.” Any mix of “not reported” and “unable to be determined” led to a coding of “unable to be determined.”

### Severity Coding Inter-Rater Reliability

Seventy-three articles (20% of the full text sample) were randomly selected for coding by a second rater, a research assistant with extensive experience with the severely affected ASD population, comparable to that of the first rater. Four dimensions of the severity coding were repeated by the second rater: evidence of inclusion of the severely affected based on each of the three domains (communication ability, cognitive functioning, and adaptive functioning), as well as overall inclusion of the severely affected population. Only two discrepancies were found between raters on a study item: one in the cognitive domain and the other in the communication domain, resulting in two discrepancies in overall inclusion. These discrepancies were resolved by an independent third rater (M.S.) who has extensive experience in the assessment and treatment of severely affected children with autism spectrum disorder. Cohen’s kappa was then calculated to determine the overall level of agreement between the two research assistant raters. Inter-rater reliability was found to be κ = 0.952 (95% CI 0.885–1.019), *p* < 0.001, indicating an almost perfect agreement between the two raters (Landis and Koch 1977).

### Analysis

Data abstracted from each article were entered into IBM SPSS Statistics 24 for analysis. Descriptive statistics in the form of frequencies (number, percent) were calculated for categorical data. Binary logistic regressions were used to evaluate trends in inclusion over time and examine predictors of inclusion. Studies using severity measures with standardized scoring metrics (e.g., IQ measures with a standardized mean of 100 and standard deviation of 15) were examined through a second analysis. The average severity scores for each ASD sample were converted into z-scores, and publication year was regressed onto average severity using meta-regression. Meta-regression was further used to determine if trends in average severity by time varied as a function of severity metric. Lastly, because older children who are more severely affected may be perceived as more challenging to study, we examined if the mean age of the sample moderated associations between average severity of the sample and publication year.

## Results

### General Findings

#### Participant Demographics

All 367 studies sampled males, while 335 studies included females. Of studies that sampled both sexes, only two had equivalent numbers of males and females, and two studies had a larger sample of females than males. Male study samples ranged in size from 2 to 376 participants, while female sample sizes ranged from 0 to 73. The mean age of the samples ranged from 1.5 to 16 years for both males and females.

#### Study Design

Nearly half (*n* = 182, 49.6%) of the studies assessed were randomized controlled trials, and the remainder were either uncontrolled trials (*n* = 100, 27.2%), controlled trials (*n* = 59, 16.1%), or case series (*n* = 26, 7.1%). With regard to type of intervention, approximately half (*n* = 186, 50.7%) of the studies were pharmacological, with smaller numbers of social/communication interventions (*n* = 50, 13.6%), complementary/alternative treatments (*n* = 43, 11.7%), and behavioral interventions (*n* = 33, 9.0%). The majority of studies targeted improvement in core ASD symptoms (*n* = 212, 57.8%), followed by cognitive/executive functioning (*n* = 39, 10.6%) and aberrant behavior (*n* = 37, 10.1%). The complete breakdown of treatment types and targets is presented in Table 1.

**Table 1:** Description of treatment study designs, including type of study and intervention type and target

	N	Percent
*Type of study*		
Randomized controlled trial (RCT)	182	49.6
Uncontrolled trial (UT)	100	27.2
Controlled trial (CT)	59	16.1
Case series (CS)	26	7.1
*Type of intervention*		
Pharmacological	186	50.7
Social/communication	50	13.6
Complementary/alternative	43	11.7
Behavioral	33	9.0
Early intervention	15	4.1
OT/sensory	14	3.8
Parent/home-based	12	3.3
Neurological	8	2.2
(Combination of interventions)	6	1.6
*Target of intervention*		
Core ASD symptoms	212	57.8
Cognitive/executive functioning	39	10.6
Aberrant behavior	37	10.1
ADHD symptoms	18	4.9
Biological/physical symptoms	17	4.6
OT/sensory problems	11	3.0
Sleep	11	3.0
Anxiety	10	2.7
OCD symptoms	2	0.5
(Multiple targets)	10	2.7

### Severity-Related Findings

#### General Inclusion of the Severely Affected

Overall, there was evidence of inclusion of the severely affected in about half of the studies evaluated (*n* = 186, 50.7%), when utilizing our liberal definition of inclusion (severity criteria met in at least one of the three domains). The remaining studies either provided clear evidence that the severely affected population was *not* included (*n* = 78, 21.3%), or that inclusion of the severely affected was unable to be determined from the information provided (*n* = 103, 28.1%). Studies of pharmacological treatments were almost three times more likely to include severely affected individuals relative to other treatment types (OR 2.85, p < 0.001; 95% CI 1.63–5.00).

#### Reporting of Severity Domains

Level of cognitive functioning was reported in nearly two-thirds of papers (*n* = 241, 65.7%), making it the most frequently reported of the three severity domains. Communication ability was reported less than half the time (*n* = 155, 42.2%), adaptive functioning (*n* = 80, 21.8%) was the least frequently reported, and the majority of papers failed to report information from either of these two domains.

#### Number of Severity Domains Reported

**Fig. 1:**
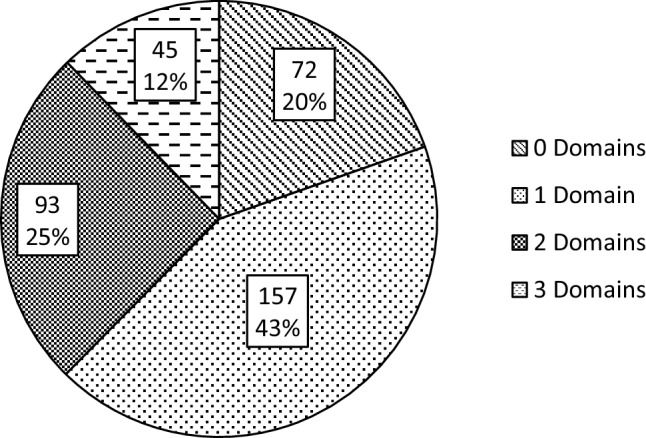
Number of severity domains (communication ability, cognitive functioning, adaptive functioning) reported in a sample of 367 ASD treatment studies

The assessed studies most frequently reported only one of the three severity domains (*n* = 157, 42.8%), with fewer studies reporting data from two (*n* = 93, 25.3%) or all three (*n* = 45, 12.3%) of the domains. Notably, nearly one in five of these peer-reviewed intervention studies in autism (*n* = 72, 19.6%) failed to report any quantitative or qualitative measure of communication ability, cognitive functioning, or adaptive ability (Fig. 1).

#### Measures Used to Report Cognitive Functioning (IQ/ID)

A total of 30 different measures were used to report level of cognitive functioning across 151 studies. An additional 90 studies reported cognitive functioning, but did not specify the measure used to assess cognitive functioning, representing 37.3% of the 241 total studies reporting participants’ cognitive functioning. Among the studies that did specify a measure, the most commonly used measure was the WISC (Wechsler 2003) (*n* = 59, 39.1%), followed by the Leiter (Roid and Miller 2011) (*n* = 28, 18.5%), and the Mullen (Mullen 1995) (*n* = 28, 18.5%). The full list of measures can be found in Table 2.

**Table 2:** Measures used to report cognitive functioning in treatment studies of children with ASD

Measure name	N	Percent
Wechsler Intelligence Scale for Children (WISC)	59	39.1
Leiter International Performance Scale	28	18.5
Mullen Scales of Early Learning	28	18.5
Stanford–Binet Intelligence Scale	17	11.3
Bayley Scales of Infant Development (BSID)	16	10.6
Wechsler Preschool and Primary Scale of Intelligence (WPPSI)	14	9.3
Wechsler Abbreviated Scale of Intelligence (WASI)	10	6.6
Griffiths Mental Development Scales	9	6.0
Differential Ability Scales (DAS)	7	4.6
Wechsler Adult Intelligence Scale (WAIS)	7	4.6
Psychoeducational Profile	6	4.0
Cattell Culture Fair Intelligence Test (CFIT)	3	2.0
Kaufman Brief Intelligence Test (KBIT)	3	2.0
Merrill-Palmer Scales of Mental Tests	3	2.0
Snijders-Oomen Non-verbal Intelligence Test	3	2.0
Gesell Developmental Schedules	2	1.3
Kaufman Assessment Battery for Children	2	1.3
Peabody Picture Vocabulary Test (PPVT)	2	1.3
Raven’s Progressive Matrices	2	1.3
Stutsman IQ test	2	1.3
Tanaka-Binet Intelligence Scale	2	1.3
Transdisciplinary Play-Based Assessment	2	1.3
Autism Treatment Evaluation Checklist (ATEC)	1	0.66
Naglieri nonverbal ability test	1	0.66
Preschool Performance Scale	1	0.66
Slosson intelligence test	1	0.66
Test of Nonverbal Intelligence (ONI)	1	0.66
Uzgiris-Hunt Scales	1	0.66
Vineland Adaptive Behavior Scales	1	0.66
Woodcock–Johnson Tests of Cognitive Abilities	1	0.66

#### Measures Used to Report Communication Ability

In total, 32 different measures were used to report communication ability across 109 studies. This represents the greatest variability in measures for the three severity domains. An additional 46 studies reported communication information, but did not specify the measure used to assess communication, representing 29.7% of the 155 studies reporting communication ability. Among the studies that did specify a measure, the most commonly used measure was the Communication subscale of the Vineland (Sparrow 2011) (*n* = 52, 47.7%), followed by the ADOS (*n* = 10, 9.2%), and the ADI-R (Le Couteur 2003) (*n* = 7, 6.4%). The full list of measures can be found in Table 3.

**Table 3:** Measures used to report communication ability in treatment studies of children with ASD

Measure name	N	Percent
Vineland Adaptive Behavior Scales	52	47.7
Autism Diagnostic Observation Schedule (ADOS)	10	9.2
Autism Diagnostic Interview (ADI-R)	7	6.4
Preschool Language Scales (PLS)	6	5.5
Peabody Picture Vocabulary Test (PPVT)	6	5.5
Expressive/Receptive One-Word Picture Vocabulary Test	5	4.6
Autism Treatment Evaluation Checklist (ATEC)	4	3.7
Reynell Developmental Language Scales	4	3.7
Comprehensive Assessment of Spoken Language (CASL)	3	2.8
Mullen Scales of Early Learning	3	2.8
PDD Behavior Inventory (PDD-BI)	3	2.8
Childhood Autism Rating Scales (CARS)	2	1.8
Children’s Communication Checklist (CCC)	2	1.8
MacArthur-Bates Communicative Development Inventories	2	1.8
Social Communication Questionnaire (SCQ)	2	1.8
Sequenced Inventory of Communication Development	2	1.8
Transdisciplinary Play-Based Assessment	2	1.8
Adaptive Social Skills Measure	1	0.92
Alpern-Boll Developmental Profile	1	0.92
Ankara Developmental Screening Inventory	1	0.92
British Picture Vocab Test	1	0.92
Communication and Symbolic Behavior Scale	1	0.92
Developmental Profile II	1	0.92
Expressive Vocabulary Test	1	0.92
Gilliam Autism Rating Scale (GARS)	1	0.92
Gesell Developmental Schedules	1	0.92
Hong Kong-Based Adaptive Behavior Scales	1	0.92
OSCC	1	0.92
Parents’ Rating Questionnaire	1	0.92
Rescorla Language Development Survey	1	0.92
Ritvo-Freeman Real Life Rating Scale	1	0.92
Social Responsiveness Scale (SRS)	1	0.92

#### Measures Used to Report Adaptive Functioning

In total, only seven different measures were used to report adaptive functioning across 80 studies, and a measure was specified in all studies that reported adaptive functioning. The most commonly used measure was overwhelmingly the Vineland (*n* = 72, 90.0%), followed by the BASC (Kamphaus and Reynolds 2007) (*n* = 3, 3.8%), and the PDD-BI (Cohen et al. 2003) (*n* = 2, 2.5%). The full list of measures can be found in Table 4.

**Table 4:** Measures used to report adaptive functioning in treatment studies of children with ASD

Measure name	N	Percent
Vineland Adaptive Behavior Scales	72	90
Behavior Assessment System For Children (BASC)	3	3.8
PDD Behavior Inventory (PDD-BI)	2	2.5
Adaptive Behavior Assessment System (ABAS)	1	1.3
Adaptive Social Skills Measure	1	1.3
Gesell Developmental Schedules	1	1.3
Hong Kong-based Adaptive Behavior Scales	1	1.3

#### Inclusion of the Severely Affected Over Time

**Fig. 2:**
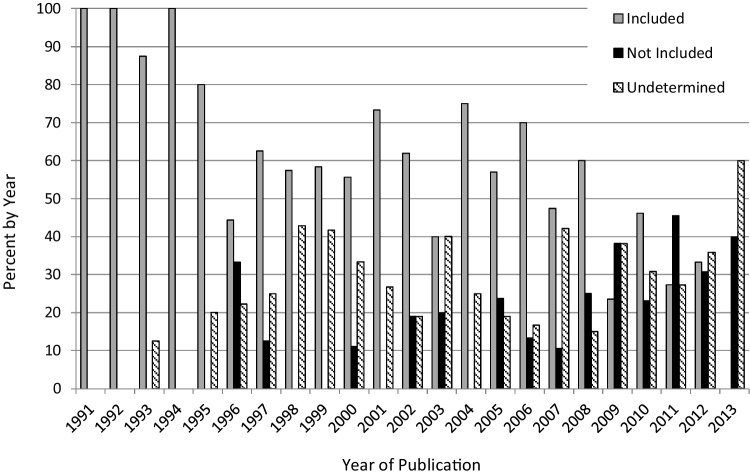
Inclusion of the severely affected ASD population in treatment studies over time

Binary logistic regression revealed that each subsequent year during the study time period, from 1990 to 2013 was associated with a 16.5% decrease in the likelihood that severely affected children would be included in ASD treatment studies (OR 0.84, p < 0.001; 95% CI 0.78–0.89) (Fig. 2; Table 5).

**Table 5:** Inclusion of the severely affected ASD population by type of intervention

Type of intervention	Severely affected included?			Total
	Yes	No	Undetermined	
	*N*(%)	*N*(%)	*N*(%)	
Pharmacological	104 (55.9)	24 (12.9)	58 (31.2)	186
Social/communication	19 (38)	21 (42)	10 (20)	50
Complementary/alternative	15 (34.9)	10 (23.3)	18 (41.9)	43
Behavioral	15 (45.5)	14 (42.4)	4 (12.1)	33
Early intervention	14 (93.3)	0 (0)	1 (6.7)	15
OT/sensory	6 (42.9)	3 (21.4)	5 (35.7)	14
Parent/home-based	8 (66.7)	0 (0)	4 (33.3)	12
Neurological	1 (12.5)	6 (75)	0 (12.5)	8
Combination of interventions	4 (66.7)	0 (0)	2 (33.3)	6

#### Average Severity of ASD Samples Over Time

**Fig. 3:**
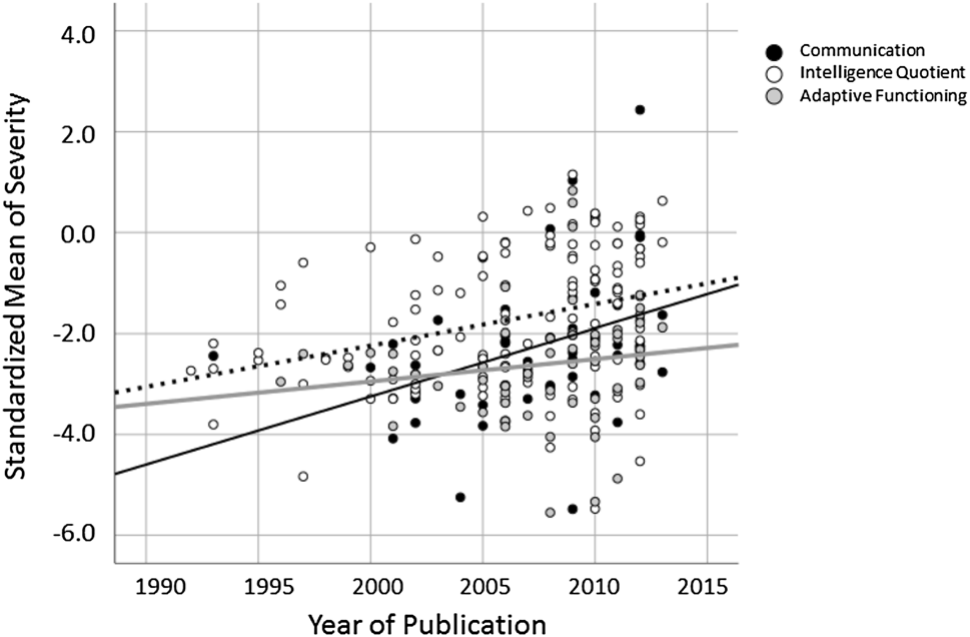
Standardized mean severity score of each sample in three domains over time. The magnitude of the association between sample severity by publication year is greatest for the communication domain, but there was no statistically significant difference between the regression lines for each domain

Standardized z-scores for average severity of communication ability, cognitive functioning, and adaptive functioning could be calculated for 64, 122, and 61 studies, respectively. Significant linear relationships were seen between publication year and both communication skills and IQ, such that the average communication skill level and IQ of ASD samples has increased with time since the early 1990s (β = 0.048, *p* < 0.001 and β = 0.033, *p* < 0.001 for time by communication and time by IQ associations, respectively). There was no association between publication year and average adaptive functioning level of ASD samples (β = 0.018, *p* = 0.192). The magnitude of association between publication year and average severity of the sample was strongest for communication skill severity; however, the association between time and the average severity of the sample was not moderated by the type of severity metric (β=-0.330, *p* = 0.126). Similarly, average sample age did not moderate associations between publication year and the average severity of the sample (Fig. 3).

## Discussion

The aim of the present study was to assess representation of the severely affected autism phenotype in the autism treatment literature. By examining the characteristics of treatment studies of children with ASD in a multistep literature analysis, we were able to discern some of the ways in which severity has been conceptualized and defined over time, as well as changes in measurement and inclusion. Our objectives were two-fold: to assess the inclusion of those severely affected by ASD in the literature over time, and to examine the ways in which severity is measured and reported utilizing three prominent and clinically relevant domains: communication ability, cognitive functioning, and adaptive functioning.

For study demographics, design and type, our results are largely consistent with the overall landscape of autism research as a whole. Male participants outnumbered females, reflecting the higher prevalence of males in ASD (Centers for Disease Control and Prevention 2018). In examining study design characteristics, the preponderance of randomized controlled trials and pharmacological studies was notable. Nearly half of the 367 articles assessed were RCTs, with a similar proportion of studies categorized as pharmacological interventions. There has been a significant increase in the publication of RCTs in the autism literature since 2008, though fewer than 10% of the trials had ≥ 100 participants (Tromans and Adams 2018). Across autism research, small sample sizes may lead to overestimation of treatment effects or overstatement of results. In considering this within the context of the present study, the same cautions should be applied to the measurement and representation of ASD severity. While the information that has been abstracted from this body of treatment studies has begun to paint a clearer picture of the landscape of autism research, several factors, including small sample sizes and variability in reporting of severity domains, should be taken into account when considering these results within the context of autism research in its entirety.

We first posited that the inclusion of the severely affected population in autism research has decreased over time. This hypothesis is supported by prior research on the topic (Bebko et al. 2008; Crosland et al. 2013), but our approach confirmed this trend through analysis of a much wider breadth of publications and a novel, more liberal definition of severity. At the outset, we developed our hypothesis of decreased inclusion based on several factors, including: possible changes in recruitment practices giving researchers access to a wider swath of the ASD population, an increase in the identification and prevalence of ASD, and increased recognition of the heterogeneity of the autism spectrum. Consistent with previous literature, the present analysis provided further evidence of a significant decrease in the inclusion of the severely affected population over time and average sample age did not moderate this association. Though severely affected subjects were included in about half of the studies evaluated, binary logistic regression revealed that each subsequent year during the study time period from 1990 to 2013 was associated with a 16.5% decrease in the likelihood of inclusion. Nearly all early treatment studies included severely affected participants, while only approximately one-third did so most recently When examining the average severity level of each sample across the domains of communication ability, cognitive functioning, and adaptive functioning, communication ability showed the strongest association with time (i.e., average communication skills of ASD samples in treatment studies increased over time). This was followed closely by average sample IQ which showed the same pattern. Finally, mean sample age did not moderate the association between average severity of the sample and publication year, suggesting that time trends in the average severity of ASD samples were independent of sample age.

A number of factors may have contributed to this decreased inclusion of severely affected participants in treatment studies. Most notably, the rapid growth in identification of the higher functioning or less severely affected portion of the autism spectrum has caused the proportion of severely affected individuals in the total ASD population to decrease markedly (Matson and Kozlowski 2011). These changes are associated with changes to the ASD diagnostic criteria in DSM IV (American Psychiatric Association 1991), DSM-IV-TR (American Psychiatric Association 2000) and DSM-5 (American Psychiatric Association 2013). Additionally, it is possible that the large increase in the higher functioning portion of the autism spectrum has expanded the range and number of modalities appropriate for intervention research, such as cognitive behavioral therapy for anxiety or anger management in ASD, which subsequently would increase the number of intervention studies that do not include the severely affected. Finally, it may be that as funding for research in autism has increased, and as more investigators are drawn to the field, they may be less apt to be familiar, have access to, or be comfortable with investigating more severely affected individuals.

In terms of the measurement and representation of severity, we hypothesized that communication ability would be the least frequently reported of the three severity domains and have the greatest variability in measures used. Adaptive functioning, however, was revealed to be the least frequently reported, with just 22% of papers including data about this domain. One possible explanation for this could involve the implementation of the Vineland II, the measure used to report adaptive functioning in 90% of studies that did so. This measure was not released until 2005 (Sparrow 2011), and the articles assessed for the present analysis were published from 1990 to 2013. Thus a large proportion of the studies were conducted prior to the implementation and proliferation of the Vineland II, which may have contributed to the substantial underreporting of adaptive functioning in the assessed studies as a whole. Regardless, the close relationship of adaptive functioning and core ASD symptoms (Frost et al. 2017) underscores that adaptive functioning is critical to the understanding of ASD severity and may serve as a beneficial proxy when describing a sample within a research context.

While our findings failed to support the first component of that second hypothesis, the second component was supported, in that communication measures were the most variable (32 unique communication measures, as compared to 30 and 7 for cognitive and adaptive functioning, respectively). This finding was unsurprising, given the well-established challenges of assessing communication in children with ASD, and the lack of a gold standard for communication assessment that is valid across the full range of the autism spectrum. The breadth of behavioral challenges and social communication deficits associated with an ASD diagnosis can contribute to difficulty in administering assessments. These challenges are further complicated by the lack of an agreed-upon definition for categorization of verbal ability in ASD. In addition, while the categorization of “minimally verbal,” for example, can range from no spoken words to perhaps 20 or 30, it fails to address abilities in receptive language or alternative communication modalities (Tager-Flusberg and Kasari 2013; Kasari et al. 2013). It is rather stunning that such lack of agreement and variability exists in a field studying a disorder where one of the two core deficits is social communication.

The finding that even in a sample of intervention studies where greater than half are RCTs, and therefore can be expected to be methodologically more rigorous, only 109 (29.7%) of the 367 studies reported a communication measure is also striking. Unfortunately, this practice is not improving over time, as evidenced by the time trends discussed previously. It does appear that some steps are being taken, however, to make progress in the field’s understanding of how to best assess communication in autism. The Interagency Autism Coordinating Committee (IACC) has highlighted the dearth of knowledge regarding nonverbal children with ASD, and the National Institutes of Health (NIH) held a workshop in 2010 to discuss these knowledge gaps and how to best address them through research (Tager-Flusberg and Kasari 2013). Promulgation of a set of standards for measurement and reporting in ASD intervention research, and perhaps ASD clinical research in general, would likely be of great benefit to the field.

## Limitations

Our findings were limited by a number of factors, most prominently by the fact that the analysis focused on a relatively narrow portion of the autism literature—treatment studies of pediatric subjects—which may reduce the generalizability to adult or non-intervention research. We chose to focus on youth as that has been the historical emphasis of the field, and we were interested in trends over time. We also studied the intervention literature because we felt it was most proximal to the clinical relevance of severity in ASD. Because the treatment literature contained so many studies of pharmacological interventions, we may have inadvertently drawn a portrait of a certain type of study and its methodologies. Even if that is the case, those patterns of severity inclusion would have been communicated to the field as a whole, reinforcing perspectives on sampling and the population as a whole. We did not include studies with only a single participant, thereby excluding the relatively large number of single-subject design publications from the behavioral treatment literature. While inclusion of the severely affected portion of the autism spectrum in that literature is of interest, we sought to maximize our evaluation of studies with the largest total sample while reducing our coding burden where feasible, and thus eliminated studies with a sample size of just one participant. In so doing, we may have missed some important information from the applied behavioral analysis literature that may have further informed the picture of severity in treatment studies. Another limitation may arise from the coding process. While detailed information was abstracted from each article as available, variability in the reporting of information may have decreased the accuracy of our assessment of the severity of each study’s sample. As an example, a prior analysis of the autism literature (Crosland et al. 2013) cited indistinct descriptions of cognitive function and communication ability as a contributing factor to decreased interrater reliability in those areas. For the present study, however, our interrater reliability calculation indicated an almost perfect level of agreement between the two raters. This likely related to the use of clear operational definitions for the coding of each severity domain. We sought to ensure the raters were being as objective as possible, while remaining cognizant of the possibility that the information reported in each study may have given an incomplete picture of the severity of the sample.

## Conclusions

The autism intervention literature contains marked variability, both currently and historically, in the measurement of severity domains, particularly communication ability, and appears to include a decreasing proportion of individuals who are severely affected by even a liberal definition of severity. Variability in defining and assessing severity in autism was underscored through empirical evidence that despite decades of advancement, the field has yet to reach a consensus on how to measure severity, and indeed if even core autism features, such as communication ability, should be considered necessary to report. Promulgation of a minimum standard set of measurement domains, and perhaps favored measures, by a governing body or journal editors, would likely benefit the field by increasing the comparability of different studies’ results and improving the interpretability of the findings through a more clear and consistent description of the sample. There was a notable decrease in the inclusion of the severely affected population over time, which may reflect changes in diagnostic criteria. This severe end of the autism spectrum, for whom assessment and treatment pose a particular challenge, is arguably the least well-understood. Exclusion of this subset from intervention and other research studies could ultimately lead to an unbalanced understanding of ASD, and possibly leave behind those who arguably have the greatest morbidity and need.
